# „Gemeinsam Wandel gestalten“: Tagungsbericht vom Kongress Armut und Gesundheit 2023

**DOI:** 10.1007/s00103-023-03754-9

**Published:** 2023-08-21

**Authors:** Maren Janella, Regine Alber, Marion Amler, Julian Bollmann, Nicole Böhme, Marina Martin, Jens Hoebel

**Affiliations:** 1 Geschäftsstelle Berlin, Gesundheit Berlin-Brandenburg e. V., Berlin, Deutschland; 2 Fachgebiet Soziale Determinanten der Gesundheit, Robert Koch-Institut, Nordufer 20, 13353 Berlin, Deutschland

Der Kongress Armut und Gesundheit gehört zu den größten regelmäßig stattfindenden Public-Health-Veranstaltungen in Deutschland. Er versteht sich als eine Plattform für den Austausch zwischen Wissenschaft, Praxis, Zivilgesellschaft und Politik. Rahmendes Thema dabei ist der Zusammenhang zwischen Armutslagen und Gesundheit. Dieser ist mittlerweile auch für Deutschland umfassend und auf breiter Datengrundlage dokumentiert. Die Befunde zeigen mit großer Übereinstimmung, dass Menschen mit einem niedrigen sozioökonomischen Status auch hierzulande deutlich häufiger von gesundheitlichen Beeinträchtigungen und schwerwiegenden chronischen Erkrankungen betroffen sind als jene mit höherem sozioökonomischen Status [1]. Zudem empfinden Menschen aus sozioökonomisch schlechter gestellten Gruppen ihren eigenen Gesundheitszustand auch subjektiv als deutlich schlechter [2]. Dabei besteht diese gesundheitliche Ungleichheit bereits im frühen Kindes- und Jugendalter, in welchem wichtige Weichen für ein gesundes Aufwachsen und Älterwerden gestellt werden [3, 4]. Letztlich spiegelt sich der Zusammenhang von Armut und Gesundheit auch in der Sterblichkeit und Lebenserwartung wider. So haben armutsgefährdete Männer im Vergleich zu Männern mit hohem Einkommen in Deutschland eine um 8,6 Jahre kürzere Lebenserwartung. Bei Frauen beträgt dieser Unterschied 4,4 Jahre [5].

Der 28. Kongress Armut und Gesundheit fand unter dem Motto „gemeinsam Wandel gestalten“ in 2 Etappen statt: Teil 1 wurde – mit über 70 Veranstaltungen – am 06. und 07.03. digital umgesetzt, Teil 2 am 21. und 22.03. in Präsenz an der Freien Universität Berlin – mit knapp 40 Veranstaltungen^1^. Insgesamt nahmen über 2000 Personen am Kongress teil, davon mehr als 500 inhaltlich Beteiligte, d. h. Moderierende oder Referierende. Vielfältige Netzwerkmöglichkeiten erlaubten, sowohl im digitalen Raum wie auch in Präsenz, neue Kontakte zu knüpfen und bestehende zu stärken.

Im Vorfeld des Kongresses fand am 03.03.2023 die Satellitenveranstaltung unter dem Motto „Der ÖGD in der kommunalen Landschaft der Zukunft“ statt:^2^ eine Veranstaltung von Gesundheit Berlin-Brandenburg e. V. in Kooperation mit der Akademie für Öffentliches Gesundheitswesen (AÖGW). Die Veranstaltung wurde im Rahmen des Kooperationsverbundes Gesundheitliche Chancengleichheit mit Unterstützung der Bundeszentrale für gesundheitliche Aufklärung (BZgA) organisiert und mit mehr als 300 Teilnehmenden umgesetzt.

## „Gemeinsam Wandel gestalten“

In insgesamt 110 Fachveranstaltungen wurde diskutiert, welche Transformationen vor dem Hintergrund aktueller Krisen möglich und notwendig sind. Dabei wurde Bezug sowohl auf das Pandemiegeschehen als auch auf den Angriffskrieg Russlands auf die Ukraine und die Klimakrise genommen. Ein Kernergebnis der Diskussionen benannte Dr. Claudia Hövener: „Es bedarf einer Public Health-Strategie und -Struktur für Deutschland, die auch in Krisenfällen resilient ist und die die besonderen Belange von Menschen in benachteiligter sozialer Lage miteinschließt.“

## Soziale Gerechtigkeit und Umweltschutz nicht gegeneinander ausspielen

Die Krisen der letzten Jahre laufen parallel und verschärfen sich teilweise gegenseitig. „All das macht ein Umdenken notwendig, aber vor allem auch einen Sprung nach vorne in die Transformation“^3^, formulierte es Maike Voss in der Eröffnungsveranstaltung.

Die Transformationsforscherin Prof. Dr. Maja Göpel ging in ihrer Keynote auf ökologische Notwendigkeiten, Zielkonflikte und Lösungsvorschläge ein. Soziale Gerechtigkeit und ökologische Schutzfrage dürften nicht gegeneinander ausgespielt werden, stattdessen müsse das aktuelle ökonomische Paradigma infrage gestellt werden. Sie führte zudem aus, dass „Mittel zum Zweck, wozu auch wirtschaftliches Wachstum gehört, irgendwie zum Ziel an sich geworden sind“. Das eigentliche Ziel sei jedoch menschliches Wohlergehen. Um diesem Ziel gerecht zu werden, seien neue Allianzen und Narrative notwendig. Umfassendere Bilanzierungen, in denen nicht nur der monetäre Umsatz, sondern auch ökologische und soziale Aspekte – sogenannte versteckte Kosten – abgebildet werden, hätten gezeigt, dass ökologischer Schutz und soziale Sicherheit keine Zielkonflikte seien, sondern sich gegenseitig fördern und transformatives Potenzial freisetzen könnten. „Es ist nicht die Summe des Geldes, das in einer Gesellschaft zirkuliert, sondern die Summe der verfügbaren Lösungen, die das Wohlergehen garantieren.“ Sie schloss ihren Vortrag damit, dass es wertschätzender gesellschaftlicher Gesprächsräume bedürfe, in denen mutig Grundsätzliches infrage gestellt und unterschiedliche Positionen und Perspektiven gehört werden, um zu gemeinsamen Lösungen zu kommen. In der anschließenden Diskussion konstatierte Frau Dr. Karin Geffert, dass das Zukunftsforum Public Health diesen Diskussionsprozess mit dem „Call for and to Action: Klimawandel und Public Health“ bereits angestoßen habe [6]. Dieser solle innerhalb der Public-Health-Community zur Entwicklung eines gemeinsamen Bewusstseins beitragen und nach außen die Expertise zum Thema aufzeigen. Dr. Ute Teichert betonte die koordinierende Rolle des Bundesministeriums für Gesundheit bei dieser Transformation.

## Wie lässt sich sozialer Wandel gestalten?

In der Veranstaltung „Sozialen Wandel gestalten: Möglichkeitsräume für Veränderungen im Gesundheitswesen erkennen und nutzen“ wurde ebenfalls grundsätzlich diskutiert, wie Transformation gelingen kann.

„Was können wir überhaupt imaginieren? Alles, was wir uns nicht vorstellen können, können wir nicht leben, ohne ein inneres Bild, … etwas, das uns auch in die Zukunft zieht“^4^, so Prof. Dr. Susanne Maria Weber, die in ihrem gemeinsamen Vortrag mit Marlena van Munster eine wissenschaftliche Einordnung der Thematik aus organisationspädagogischer Perspektive vornahm. Unter anderem stellte sie den Wert von Partizipation, Vernetzung, Zukunfts- und Innovationslaboren sowie Co-Kreation in realen Kontexten für die Gestaltung von Wandel heraus.

Zentrales Problem bei der Gestaltung sozialen Wandels sei die Diskrepanz zwischen Wissen und Handeln, auf die Marlena van Munster näher einging. Sie zeigte die Notwendigkeit auf, multiprofessionelle sowie intersektorale Zusammenarbeit zu stärken und einen gemeinsamen normativen Rahmen zu entwickeln, um Veränderungen zu ermöglichen. Hierfür brauche es entsprechende organisationale Strukturen, die auf die Vernetzung abzielen, so van Munster. Die Parkinson-Netzwerke seien hier ein Beispiel guter Praxis. Sie stellte im weiteren Verlauf das Vorgehen und den Nutzen von Netzwerken dar. Anhand dessen wurden Implikationen für sozialen Wandel aufgezeigt und anschließend mit den Teilnehmenden diskutiert. Prof. Dr. Weber hob darin auch den Kongress selbst als Positivbeispiel für Netzwerkbildung hervor.

## Die Pandemie als Brennglas für Ungleichheiten

Die COVID-19-Pandemie hat die sozialen und gesundheitlichen Ungleichheiten in Deutschland wie unter einem Brennglas sichtbar gemacht. Waren ganz am Anfang der Pandemie sozial privilegierte Gruppen häufiger mit dem damals noch neuartigen Coronavirus infiziert, so war ab dem Winter 2020/2021 ein entgegengesetzter sozialer Gradient erkennbar: Menschen in sozioökonomisch schlechter gestellten Regionen erkrankten und verstarben häufiger an bzw. mit COVID-19 [7].

Kolleg*innen des Projektes INHECOV (Socioeconomic Inequalities in Health during the COVID-19 Pandemic), einem Verbundprojekt von Robert Koch-Institut (RKI), Uniklinikum Düsseldorf und der Universität zu Köln, stellten hierzu aktuelle Forschungsergebnisse in der jährlich auf dem Kongress stattfindenden Fachforen-Reihe „Soziale Determinanten“ vor und zur Diskussion. Die Ergebnisse zeigen, dass es im Infektionsgeschehen eine wichtige Rolle spielte, welche Möglichkeiten die einzelnen Menschen hatten, die vorgeschriebenen oder empfohlenen Maßnahmen zum Infektionsschutz umzusetzen. Menschen aus geringer qualifizierten und statusniedrigeren Berufsgruppen hatten beispielsweise weniger Gelegenheit, ihre beruflichen Kontakte zu reduzieren und ins Homeoffice zu wechseln [8]. Je nach beruflichem Anforderungsniveau war auch das COVID-19-Erkrankungsrisiko ungleich verteilt – zu Ungunsten von Berufstätigen ohne Leitungsfunktionen und statusniedrigeren Berufsgruppen mit helfenden Tätigkeiten [9]. Ökonomische Sorgen, die allgemein dazu beitragen, dass Menschen in Erwerbsarmut eine schlechtere psychische Gesundheit aufweisen als regulär Beschäftigte, stiegen während der Pandemie unter Beschäftigten insgesamt an, wie Ergebnisse des Projekts zeigen.

In einem weiteren Fachforum zur COVID-19-Pandemie wurde auf Bedingungen im Zusammenhang mit Migration und Flucht geschaut. Menschen mit Fluchterfahrungen in Sammelunterkünften waren einem besonderen Risiko für eine SARS-CoV-2-Infektion ausgesetzt, da sie wenig bis keine Möglichkeiten hatten, sich an die vorgegebenen Hygienemaßnahmen zu halten [10]. Menschen mit eigener Migrationserfahrung wiesen zudem eine um 10 Prozentpunkte niedrigere COVID-19-Impfquote auf [11].

Die jährlich auf dem Kongress stattfindende Veranstaltung „Health Inequalities“ richtete in diesem Jahr den Fokus auf Ungleichheiten in der Kinder- und Jugendgesundheit während der Pandemie. Für Kinder und Jugendliche zwischen 12 und 15 Jahren waren die Einschnitte durch die Coronapandemie am gravierendsten, betonte Prof. Dr. Klaus Hurrelmann im Gespräch mit Dr. Irene Moor. „Es könnte sein, dass die Coronapandemie ein relevantes Ereignis für diejenigen geworden ist, die zwischen 12 und 20 diese Pandemiephase erlebt haben, und dass das Spuren hinterlässt“, so Hurrelmann. Er nannte auch Ergebnisse der COPSY-Studie (Corona und Psyche), die zeigen, dass das Anspannungs- und Belastungsniveau in der Pandemie auf das Doppelte gestiegen ist und Kinder und Jugendliche aus Familien mit niedrigem sozioökonomischen Status auch hier besonders betroffen sind [12]. Höhere Belastungen unter Kindern und Jugendlichen in sozioökonomisch benachteiligten Lagen zeigten auch der Präventionsradar der DAK-Gesundheit, Auswertungen der Düsseldorfer Schuleingangsuntersuchung und die Studie „Kindergesundheit in Deutschland aktuell“ (KIDA) des RKI, die in dieser Session vorgestellt wurden. In zukünftigen Pandemieplanungen müssten die sozialen Determinanten als Befund mit aufgenommen werden. Darüber hinaus sei ein Monitoring nötig, um frühzeitig zu erkennen, wer besonders gefährdet und/oder betroffen ist. Diese Personengruppen müssten in die Planung von Maßnahmen mit einbezogen werden, so Dr. Claudia Hövener in der Veranstaltung „Migration und Flucht, Fokus COVID-19“.

## Krieg und Flucht

Der andauernde Angriffskrieg auf die Ukraine war auch dieses Jahr wieder Thema auf dem Kongress. Neben kriegerischen Konflikten bewegen aber auch andere Motive Menschen zur Flucht. Der Weg und die Ankunft vor Ort gestalten sich oftmals sehr schwierig [13, S. 13 ff.]. Niklas Nutsch und Andreas Gold, Universität Bielefeld, führten aus, dass Migrant*innen bei Ankunft und vor Asylantragsstellung nach §§ 4 und 6 Asylbewerberleistungsgesetz nur sehr eingeschränkt Zugang zu Gesundheitsleistungen hätten. Bei einem laufenden Asylverfahren seien der Umfang der Behandlungen und die Art und Weise, wie diese gewährt werden, abhängig davon, in welchem Bundesland sie sich befinden. Erst nach 18 Monaten erhielten Migrant*innen dann in der Regelversorgung Analogleistungen zu denen des SGB V (Fünftes Sozialgesetzbuch) und Zugang zu einer elektronischen Gesundheitskarte (eGK; [14]). Fehlende oder verzögerte Behandlung hätten dabei negative gesundheitliche Auswirkungen, mit – nicht nur ökonomisch – gravierenden Folgen. Eine flächendeckende Einführung der eGK würde nicht nur die betroffenen Menschen, sondern auch das Gesundheitssystem entlasten. Die Gesundheitskarte führe zu einer größeren Annahme von hausärztlicher Versorgung und zu einer bedarfsgerechteren Nutzung der fachärztlichen und der Notfallversorgung. Zusätzlich führe die eGK zu einer verminderten Diskriminierung und Stigmatisierung [15].

Eine stärkere finanzielle Unterstützung der Flüchtlingsarbeit und v. a. auch der Ehrenamtlichen in dem Bereich forderte Manfred Evers, Volkssolidarität Ratingen, im Panel „Arbeit mit Menschen mit Fluchthintergrund – professionell und ehrenamtlich“. Jana Spieckermann, Volkssolidarität Berlin, berichtete im Panel über die Relevanz der ehrenamtlichen Unterstützung auch in der Zusammenarbeit mit professionellen Pflegekräften, zum Beispiel in der Arbeit mit älteren Geflüchteten. Mit dem Angriffskrieg in der Ukraine kamen auch viele ältere Menschen mit ihren Familien nach Deutschland und trafen dort auf ein bereits überlastetes Pflegesystem. Jana Spieckermann berichtete von der großen Solidarität unter den Pflegenden, der trägerübergreifenden Zusammenarbeit und der Unterstützung von Ehrenamtlichen, insbesondere durch Dolmetschende.

## Planetare Gesundheit

Dass die Erde krank ist, stellte Prof. Maja Göpel in ihrer Keynote fest.^5^ Hieran schloss die Veranstaltung „Gesundheitsförderung und Prävention als Ziel der großen Transformation – was es jetzt braucht“ des Centre for Planetary Health Policy an [16]. Die Auswirkungen der ökologischen Krisen bedrohen Gesundheit und Wohlergehen und dabei überproportional sozial benachteiligte Gruppen. So stellte der Wissenschaftliche Beirat für Globale Umweltveränderungen in seinem Bericht „Planetare Gesundheit: worüber wir jetzt reden müssen“ (2021) fest, dass die Lebensweise der Menschen krank macht und den Planeten zerstört.

Gesunde Menschen könnten nur auf einem gesunden Planeten existieren. Für die planetare Gesundheit – so die Schlussfolgerung – müsste die Menschheit eine zivilisatorische Wende einleiten [17]. Katharina Wabnitz stellte hierzu die These auf, dass planetare Gesundheit erreicht sei, wenn politische, soziale und ökonomische Systeme inhärent gesundheitsfördernd und präventiv seien. Dies würde bedeuten, dass der heute „nachsorgende und kompensierende“ Sozialstaat ein „vorsorgender und nachhaltiger“ Sozialstaat werden müsse. Der Sozialstaat könnte hier als Kompass wirken, der mit klimaschützenden (lebensweltlichen) Maßnahmen auch der planetaren Gesundheit und der Chancengerechtigkeit als Co-Benefits den Weg weist [16].

Auch wenn verschiedene Fachverbände (GKV-Spitzenverband 2023, die Nationale Präventionskonferenz 2023) die Wichtigkeit von Klimaschutz- und Gesundheitsförderungsmaßnahmen anerkennen würden, fehle zur Umsetzung bislang die gesetzliche Verankerung [18].^6^ Laut Charlotte Schnitzler sei es jedoch nicht einfach, auf gesetzlichem Weg interdisziplinäre Maßnahmen der relevanten Akteur*innen festzuschreiben. Dies hänge u. a. mit den unterschiedlichen Kompetenzen von Bund, Ländern und Gemeinden zusammen. Wünschenswert sei die Ausrichtung auf einen Planetary-Health-Ansatz, der auch Einfluss auf die europäische Gesetzgebung habe. Schnitzler schlug ein zweites Artikelgesetz, parallel zum Präventionsgesetz, vor, in dem zum Beispiel Stadt- und Verkehrsplanung aufgegriffen würden. Hierdurch könne auch die Verantwortung der Krankenkassen auf weitere Akteur*innen verteilt werden. Daneben schlug Schnitzler vor, bestehende Strukturen wie das Präventionsforum, die Bundesrahmenempfehlungen und den Leitfaden Prävention zu erweitern. Dafür müssten Akteur*innen zu Planetary Health geschult und verbindliche Nachhaltigkeitskriterien in den Leitfäden etabliert werden. Zu Diskrepanzen zwischen Theorie und Praxis käme es, weil intendierte Handlungsspielräume der ausgestaltenden Akteur*innen nicht praxisnah formuliert und durchdacht wären.

Stefan Pospiech, Gesundheit Berlin-Brandenburg e. V., erklärte, dass das Präventionsgesetz als Hebel zu kurz sei, um einen großen Transformationsprozess zu initiieren, da es „nur“ die Sozialversicherungsträger verpflichte und die damit einbezogenen Summen zu wenig Handlungsspielraum böten. Prof. Dr. Raimund Geene kritisierte, dass bestehende Instrumente oft schwerfällig und langsam seien und aktives und oft kurzfristiges Engagement in partizipativen Prozessen ausbremsten. Wie konkret eine Erweiterung bestehender Instrumente aussehen kann, wurde mit dem Projekt KliGeS – Ansätze für klimagesunde Settingprävention von Luisa Stömer, Berliner Institut für Gesundheit und Sozialwissenschaft, vorgestellt. Das Zugehen auf vulnerable Gruppen und die Fokussierung auf verhältnispräventive Maßnahmen seien zentral.

Im Fachforum „Daten für Taten“ berichtete die Gesundheitsberichterstattung des Bundes am RKI, dass zu ihren aktuellen Aufgaben die Erarbeitung eines aktualisierten Sachstandberichts zum Thema Klimawandel und Gesundheit gehört, der 2023 in einer Beitragsreihe im Journal of Health Monitoring veröffentlicht wird [19].^7^ Um gesundheitsgerechten Klimaschutz und Klimaanpassung vor Ort im kommunalen Setting zu gewährleisten, sei die Verbreitung dieser Erkenntnisse von großer Bedeutung.

## Wie können Menschen mit Armutserfahrungen stärker in gesellschaftliche Prozesse einbezogen werden?

Seit 2 Jahren wird der Kongress unterstützt vom Gremium „Menschen mit Armutserfahrungen“. In diesem Jahr luden die Mitglieder dieses Gremiums ein zu diskutieren, wie Menschen mit Armutserfahrungen stärker in gesellschaftliche Prozesse einbezogen werden können. Im Nachgang zu dieser Veranstaltung wurden 10 Thesen als Ergebnis dieser Veranstaltung von den Gestalter*innen zusammengefasst.^8^ Ein wichtiger Punkt, der dabei herausgestellt wurde, ist, dass für die Selbstorganisation armutsbetroffener Menschen auf kommunaler Ebene Ressourcen und Strukturen notwendig sind.

Dr. Wolfgang Strengmann-Kuhn (Bündnis 90/Die Grünen) begrüßte das starke Engagement des Gremiums „Menschen mit Armutserfahrungen“ und ermutigte die Teilnehmenden dazu, sich zu organisieren und verschiedene Kommunikationskanäle zu nutzen, um mit Politiker*innen in Kontakt zu kommen. Ein Teilnehmer, der selbst obdachlos ist, gab zu bedenken, dass die Menschen genau dafür Ressourcen benötigen. Mindestens ein Telefon und eine Internetverbindung seien erforderlich. Für den Armuts- und Reichtumsbericht gibt es derzeit ein Gutachtergremium, in welchem wissenschaftliche Expertise interdisziplinär eingebunden sei, und einen Beraterkreis, in dem Interessenvertretungen mitwirkten, so Strengmann-Kuhn. Es fehle jedoch die Lebensweltexpertise armutsbetroffener Menschen. Förderprogramme müssten so angepasst werden, dass mehr partizipative Projekte und Selbstorganisation ermöglicht würden.

Jürgen Schneider (Armutsnetzwerk e. V.) führte aus, dass bereits in der Phase der Ideenentwicklung die Lebensweltexpertisen mit einbezogen werden sollten. Das Wort armutsbetroffener Menschen sollte genauso zählen wie die Perspektiven von Wissenschaftler*innen, so Erika Biehn (VAMV, Verband alleinerziehender Mütter und Väter Landesverband Bremen e. V.). Betont wurde auch, dass sprachliche Barrieren abzubauen sind. Wenn Bildung als Indikator für Armut benannt wird, sollte immer geprüft werden, auf welchen sprachlichen Ebenen die Themen diskutiert werden. Um armutsbetroffene Menschen einzubeziehen, werde auch finanzielle Unterstützung in Form von Aufwandsentschädigungen oder Honoraren benötigt. Es brauche schlichtweg Geld, um Armut zu bekämpfen, so Wolfgang Strengmann-Kuhn: Nur mit finanzieller Unterstützung können Bildung, Mobilität, gesellschaftliche und digitale Teilhabe für Menschen in Armut gewährleistet werden.

## „Sozialpolitik ist Demokratiepolitik“

Der Präsenzteil des Kongresses an der Freien Universität in Berlin wurde mit einer Veranstaltung eröffnet, in der die wahrnehmbaren Auswirkungen der Krisen auf unser demokratisches Gesellschaftssystem und damit auf den gesellschaftlichen Zusammenhalt benannt wurden^9^.

Bundespräsident Frank-Walter Steinmeier eröffnete diese Veranstaltung, betonte die Wichtigkeit der Datenlage der Public-Health-Wissenschaften zur gesundheitlichen Ungleichheit und die Notwendigkeit, diese kontinuierlich in die Öffentlichkeit zu tragen. In Deutschland sei Armut sichtbar, so der Bundespräsident. In der gesundheitlichen Ungleichheit sah er eine Herausforderung für unser Sozialsystem. „Sozialpolitik ist Demokratiepolitik“ und so müsse neben den Krisen auch der gesellschaftliche Zusammenhalt auf der politischen Agenda stehen.

Prof. Dr. Gerhard Trabert (Armut und Gesundheit in Deutschland e. V.) knüpfte an die Worte des Bundespräsidenten an und sprach von einer Destabilisierung der Demokratie durch die Zunahme von Armut und gesundheitlicher Ungleichheit vor dem Hintergrund aktueller Krisen. Fühlten sich Menschen nicht mehr respektvoll behandelt, könnten sie empfänglicher werden für ein Infragestellen der Demokratie durch rassistische, rechtspopulistische Strömungen. Unser demokratisches System müsse von innen geschützt werden durch die Bekämpfung von gesundheitlicher Ungleichheit und Armut sowie durch die Herstellung von Gender‑, Bildungs- und Verteilungsgerechtigkeit. Es müsse zusätzlich nach außen geschützt werden, indem der Frieden mit anderen Nationen erhalten wird. In der daran anschließenden Gesprächsrunde betonten Dr. Katharina Böhm (Hessische Arbeitsgemeinschaft für Gesundheitsförderung e. V.) und Prof. Dr. Martin Dietrich (Bundeszentrale für gesundheitliche Aufklärung) die Vernetzung als eine der wichtigen Aufgaben von Public Health, bundesweit durch den Kooperationsverbund Gesundheitliche Chancengleichheit und auf der kommunalen Ebene durch die Befähigung von Akteur*innen innerhalb bestehender Strukturen. Mit einer gemeinsamen Public-Health-Strategie für Deutschland solle das Thema der gesundheitlichen Chancengerechtigkeit in Zukunft institutionell verankert und in den Aufgaben und Arbeitsprozessen von Anfang an mitgedacht werden.

## Erstmals nach 3 Jahren wieder Austausch in Präsenz

Prof. Dr. Gerhard Trabert betonte die Wichtigkeit einer (armuts-)sensiblen Sprache. Diese Thematik wurde in einem eigenen, von der Akademie für Öffentliches Gesundheitswesen konzipierten Seminar „Words don’t come easy: Auf dem Weg zu einer gemeinsamen Sprache“ weiterdiskutiert. Selbst von Public-Health-Akteur*innen gut gemeinte Begriffe wie „sozial benachteiligt“ seien Zuschreibungen, die mitunter stigmatisierend wirkten. Dies könne dazu führen, dass Menschen Angebote ablehnten, weil sie sich nicht damit identifizierten. Im Austausch zwischen Teilnehmenden, Vertreter*innen für Praxis und Wissenschaft sowie Betroffenen wurde der Raum zur kritischen Selbstreflexion über diese Problematiken und den eigenen Sprachgebrauch eröffnet.

In einer Veranstaltung der Hilfs- und Menschenrechtsorganisation „medico international“ und des globalen Netzwerks „People’s Health Movement“ wurde der 6. alternative Gesundheitsreport „Global Health Watch 6 (GHW)“ vorgestellt und mit den Autor*innen Dr. Roman Rafael Vega Romero und Dian Maria Blandina diskutiert. Angesichts der weltweiten COVID-19-Pandemie und der globalen Klimakrise – beides markiert und verschärft bestehende Ungerechtigkeiten der neoliberalen Globalisierung – beschreibt der GHW die Bedeutung der sozialen Kämpfe für das große Ziel, Gesundheit für alle zu erreichen. Der Untertitel des Berichts „In the shadow of the pandemic“ war zugleich der Titel der Veranstaltung, die auch aufgezeichnet wurde und online abrufbar ist.^10^

Neben den großen Fragen globaler Gesundheit wurde in einer anderen Veranstaltung beispielsweise auch die urbane Gesundheit im Ruhrgebiet diskutiert. Um eine inklusive gesundheitsfördernde Stadtentwicklung zu ermöglichen, wurden im Projekt „ParStaR“ (Partizipative Methoden für StadtGesundheit Ruhr) der Hochschule für Gesundheit in Bochum Methoden entwickelt und erprobt, um Bedarfe zu erfassen und unter anderem für die Stadtverwaltung aufzubereiten. Die Methoden umfassten unter anderem den Stadt-Raum-Monitor, eine Foto-Safari und auch die Arbeit mit Lego. Die Co-Forscher*innen, die sich selbst als „Regenbogengruppe“ bezeichnen,^11^ wurden von Anfang an als Expert*innen ihrer eigenen Lebenswelt einbezogen und stellten ihre Ergebnisse selbst vor – ein absolutes Highlight des Kongresses, nicht nur für die Co-Forschenden selbst, wie Standing Ovations aus dem Publikum zeigten. Zudem wurden die Erfahrungen eines Roundtable-Verfahrens mit Teilnehmenden aus Praxis, Wissenschaft und Gesellschaft geteilt. Die Potenziale von „Health in All Policies“ wurden dabei insofern erfahrbar, als dass durch die Zusammenkunft unterschiedlicher Akteur*innen neue Allianzen gebildet wurden, in denen der Mehrwert der Zusammenarbeit greifbar wurde, so ein Fazit von Prof. Dr. Heike Köckler.

Beim Science Slam, der seit 2019 jährlich auf dem Kongress stattfindet, konnten studentische Abschlussarbeiten und Promotionsvorhaben auf unterhaltsame Weise präsentiert werden. Die Nachwuchskräfte sprachen über Zervixkarzinomprävention für Sexarbeiterinnen, jugendliche Perspektiven auf die Corona-Maßnahmen, diamorphingestützte Substitutionstherapie aus Klient*innenperspektive und digitale Gesundheitsinterventionen für Jugendliche mit Diabetes mellitus [20].

In der Abschlussveranstaltung wurden Menschen mit Armutserfahrung mit Vertreter*innen der Politik in Austausch gebracht. 16,9 % der Menschen in Deutschland leben in Armut oder sind armutsgefährdet [21]. Dennoch finden die Stimmen dieser Menschen zu selten Gehör in politischen Entscheidungsprozessen. Die Forderung: „redet mit statt nur über uns“, brachte Jürgen Schneider als Vertreter des Armutsnetzwerks e. V. in die Abschlussveranstaltung ein. Die genannten, im digitalen Teil erarbeiteten 10 Punkte wurden mit Politiker*innen diskutiert. Die Abschlussveranstaltung ist vollständig online einsehbar.^12^

Neben dem breiten inhaltlichen Programm ermöglichte das Wiedersehen in Präsenz endlich auch wieder den direkten Austausch außerhalb der Fachveranstaltungen. So gab es einen Raum, der für Vernetzungstreffen u. a. der BACK (Bundesarbeitsgemeinschaft Anonyme Behandlungsschein- und Clearingstellen für Menschen ohne Krankenversicherung) genutzt wurde. Auf dem Markt der Möglichkeiten, der den gesamten Präsenzteil begleitete, stellten sich auf 2 Etagen 32 Institutionen, Projekte und Initiativen vor. Sie luden zum unverbindlichen Austausch, zum Plausch bei einem Kaffee oder zum Stöbern am Büchertisch ein. Auch eine Suche-Biete-Börse begleitete den gesamten Kongress, sowohl digital als auch in Präsenz.

## Fazit aus dem diesjährigen Kongress und Ausblick

Der Kongress Armut und Gesundheit hat abermals gezeigt: Es gibt viele mutige Initiativen, anspruchsvolle Vorhaben und engagierte Akteur*innen. Die nach wie vor einmalige Mischung der Teilnehmenden – Praxis und Zivilgesellschaft, Wissenschaft und Politik – hat auch in diesem Jahr verdeutlicht, dass der „Multilog“ – das vielstimmige Gespräch – und der Austausch zu Problemen und Handlungsansätzen neue Perspektiven eröffnen und fruchtbare Kooperationen anstoßen können. Auch wenn die Parallelität der aktuellen Krisen viele erschöpft und verunsichert, wurden Möglichkeiten und Chancen aufgezeigt, den (Werte‑)Kompass in Richtung Wohlbefinden neu auszurichten und Paradigmen (z. B. aktuelles ökonomisches Paradigma) zu hinterfragen. Hierzu lädt der Kongress Armut und Gesundheit auch im kommenden Jahr wieder ein: Am 05. und 06.03.2024 am Henry-Ford-Bau der Freien Universität Berlin und am 12.03.2024 im virtuellen Raum – Save the Date.

## Footnotes

1 Dokumentation: https://www.armut-und-gesundheit.de/ueber-den-kongress/doku-2023; Zugegriffen 13.07.2023.

2 Satellitenveranstaltung zum Kongress Armut und Gesundheit. Freitag, den 03.03.2023. https://www.gesundheitliche-chancengleichheit.de/satellit-armut-gesundheit/satellit-2023/. Zugegriffen: 22.06.2023.

3 Eröffnungsveranstaltung Kongress Armut und Gesundheit 2023, digitaler Teil. https://www.youtube.com/watch?v=3QCiVCp063g. Zugegriffen: 22.06.2023.

4 „Auf dem Weg zu regional vernetzten Gesundheitssystemen“. Vortrag auf dem Kongress Armut und Gesundheit 2023. https://www.youtube.com/watch?v=CR0LxGFJUVQ. Zugegriffen 22.06.2023.

5 Eröffnungsveranstaltung Kongress Armut und Gesundheit 2023, digitaler Teil. https://www.youtube.com/watch?v=3QCiVCp063g. Zugegriffen: 10.07.2023.

6 GKV-Spitzenverband (2023) Leitfaden Prävention. https://www.gkv-spitzenverband.de/krankenversicherung/praevention_selbsthilfe_beratung/praevention_und_bgf/leitfaden_praevention/leitfaden_praevention.jsp. Zugegriffen: 23.06.2023.

7 Einsehbar unter: http://www.rki.de/klimabericht. Zugegriffen: 10.07.2023.

8 10 Thesen des Gremiums „Menschen mit Armutserfahrungen“. https://www.armut-und-gesundheit.de/ueber-den-kongress/gremium-menschen-mit-armutserfahrungen. Zugegriffen: 23.06.2023.

9 Eröffnungsveranstaltung Kongress Armut und Gesundheit 2023, Präsenzteil. https://www.youtube.com/watch?v=FU9KTF20fgo. Zugegriffen: 22.06.2023.

10 Die Weltkrise ist immer auch eine Gesundheitskrise. Fachforum auf dem Kongress Armut und Gesundheit. https://www.youtube.com/watch?v=deqdCNSfdwU. Zugegriffen: 23.06.2023.

11 „Wir möchten betonen, dass einige von uns Co-Forscher*innen in der Wissenschaft mit sogenannter geistiger Behinderung oder mit Lernschwierigkeiten bezeichnet werden. Wir möchten hervorheben, dass unser Team diese Bezeichnung nicht für sich selbst verwenden würde.“ Das Team hat sich daher „Regenbogengruppe“ genannt. https://www.armut-und-gesundheit.de/kongress-2024/gremium-menschen-mit-armutserfahrungen. Zugegriffen am 12.07.2023.

12 Abschlussveranstaltung Kongress Armut und Gesundheit 2023. https://www.youtube.com/watch?v=pk6mE7_yzEk. Zugegriffen: 23.06.2023.
